# What Have You Been Told? Awareness of Prognosis of Patients in an Italian Home Palliative Care Service

**DOI:** 10.1089/pmr.2024.0072

**Published:** 2025-02-10

**Authors:** Claudia Bolpagni, Federico Nicoli, Patrizia Borghetti, Matteo Rota, Giovanni Zaninetta, Michele Fortis

**Affiliations:** ^1^ Hospice and Palliative Care, Domus Salutis Clinic, Teresa Camplani Foundation, Brescia, Italy.; ^2^ Clinical Ethics Service, Domus Salutis Clinic, Teresa Camplani Foundation, Brescia, Italy.; ^3^ Department of Biotechnology and Life Sciences, Center for Clinical Ethics, University of Insubria, Varese, Italy.; ^4^ Psychology Service, Domus Salutis Clinic, Teresa Camplani Foundation, Brescia, Italy.; ^5^ Department of Molecular and Translational Medicine, Università degli Studi di Brescia, Brescia, Italy.

**Keywords:** awareness, clinical ethics, communication, home care, palliative care

## Abstract

**Background:**

In palliative care, investigating prognosis awareness is a milestone for effective and comprehensive patient intake care. The literature shows that over the past half-century, regarding prognoses, the data report less willingness to provide information, despite patients’ wishes.

**Objective:**

To investigate the varying degrees of awareness of prognosis of patients and their caregivers admitted to an Italian Palliative Home Based Care Service.

**Design:**

A monocentric observational survey study with questionnaires created by the research team and completed by physicians, caregivers, and healthcare professionals (HCPs) during the intake. The assessment of any statistically significant differences was evaluated through McNemar’s test.

**Subjects:**

Forty patients (±75 years old, 60% females) with an estimated prognosis of more than 10 days, and for whom there was an opportunity to provide informed consent, who were intake at the Home Palliative Care Service of the Domus Salutis Clinic in Italy, from January 1 to June 30, 2022 were recruited.

**Results:**

In total, 52% of patients were fully aware of their prognosis at the time of intake, although 75% had asked to be informed about their prognosis. Before death, the total percentage of patients who were aware of their prognosis was 72. Twenty percent of patients were informed of their prognosis during the course of treatment. The total number of patients aware of prognosis, from the caregiver’s perspective before death, was 28 (71%). The postmortem questionnaire revealed that the team had discussed prognoses with 86% of patients.

**Conclusion:**

Periodic re-evaluation of prognosis awareness during the course of care is essential, awareness increased significantly.

## Introduction

Awareness, in general, can be understood as *a dynamic process that each person enacts in making information his or her own, understanding it cognitively, and accepting it emotionally*.^1^ In palliative care (PC) and especially in “end-of-life” situations, prognosis awareness of the disease is a prerequisite for effective sharing of clinical and therapeutic decisions.^2–4^

Prognosis awareness is assessed by indicators such as being aware of the terminal nature of one’s disease, knowing the proximity of death and being in PC service care.^5,6^ Further, previous studies suggested that diagnosis awareness promotes communication regarding the disease prognosis.^5,7–10^

There is no consensus on which approach is most useful in clinical practice and/or research to assess prognosis awareness, due to the absence of methods which define psychometric characteristics. This precludes the possibility of assessing their reliability and validity. However, the use of structured or semi-structured instruments appears to offer greater validity than unstructured approaches.^5–7^

A recent review^8^ showed that slightly fewer than half (49.1%) of patients with advanced/terminal disease knew their prognosis. Furthermore, the same study found a marked disparity in prognosis awareness in patients with malignancies.

As reported in the literature, among European countries, Italy has the lowest prevalence of accurate prognostic awareness in light of sociocultural factors.^8^

There is a lack of studies in the literature that investigate the change in prognosis awareness along the course of care during the last month of life; the little evidence available in the literature confirms that prognosis awareness tends to remain stable over time in those patients who are already aware of their situation during the last weeks of life.^8,9,11^ Few studies have shown an increase in prognosis awareness on the part of the patients who were unaware of their prognosis at intake.^12–14^

In Italian settings, communication of diagnosis and prognosis is a medical responsibility.^15,16^ Along the care pathway, HCPs may be involved by the patient and caregiver in requests for further confirmation, clarification, or in-depth information. Thus, information sharing within the HCPs team is created regarding the accompaniment to patient prognosis awareness.

The research focus of this study recognizes prognosis awareness as a key element in allowing the patient a more adequate chance to share in decision making at the end of life. This single-center study starts by underscoring the need to monitor prognosis awareness regularly in clinical practice with the administration of dedicated questionnaires even when death is imminent.

## Aim

The aim of the study was to describe the awareness of the prognosis of patients who were residents at the Home Palliative Care service of the Domus Salutis Clinic in Brescia, tracing their evolution during the care process until the exitus.

## Methods

### Study design

We performed a monocentric observational survey study of 40 patients and their caregivers and HCPs which referred to the Italian Home Palliative Care service of the Domus Salutis Clinic from January 1, 2022 to June 30, 2022. The study was approved by the Brescia Ethics Committee on May 18, 2021 (NP 4784).

### Setting

The study was carried out exclusively in patients referred to an Italian Home Palliative Care Service. This service involves the provision of care at the patient’s home by a multidisciplinary team. It is part of the specifics of the PC network provided by Italian Law n. 38 of 2010.

### Participants

Of all patients (no. 101) admitted from January to June 2022 by an Italian Home Palliative Care Service, 48 patients were excluded due to inauspicious short-term prognoses, dementia, absence of a caregiver, and in one case due to the presence of a language barrier; 10 patients were still involved in the service at the end of the study, and 3 patients had been transferred to settings other than the hospice, and therefore exited the study.

The 40 patients included in the study had an estimated prognosis of more than 10 days, and there was an opportunity for them to provide informed consent. Caregivers of patients enrolled in the study who were able to give informed consent and were indicated as primary caregivers by the patient were also recruited.

### Data collection

Within the first 7–10 days of the patient’s intake at the PC service, the patient and their caregiver were invited to participate in the study, and appropriate information and informed consent was delivered. During the intake, every 7–14 days, the administration of the questionnaires and scales below were repeated, with the aim of periodically re-evaluating the patient’s degree of awareness regarding his or her prognosis.

The research team created the questionnaires analyzing the main structured and unstructured methods of assessing prognosis awareness presented in the literature.^5^ The questionnaires were not validated; however, they can be generalizable and used at home PC services.

The physician’s questionnaire (19 items), created by the research team, investigates the degree of awareness of prognosis (items 12, 13, control 14, 15) of both the patient and the caregiver.

Therefore, to stratify prognosis awareness, closed questions were included in the questionnaire which assessed, according to the clinician: whether the patient and caregiver were fully aware of their prognosis, had not been informed but perceived their terminally ill condition, had not been informed but had unrealistic expectations, were not aware of prognosis. In the case of an affirmative answer in the first two cases, the patient was determined to be made aware of the prognosis. Two control questions were also included in the questionnaire which stratified prognosis awareness according to the patient’s expectations of his or her future and their perception of the disease (items 14–15). The ways and settings in which previous communications regarding end of life and diagnosis were made by medical staff, and the patient’s awareness of being in the care of the PC service were also explored in the questionnaire (Supplementary Appendix SA1).

The caregiver questionnaire, created by the research team, is divided into two parts. The first investigates the caregiver’s sociodemographic characteristics (age, gender, education level, caregiver’s relationship with the patient). The second part (16 items) investigates what the patient’s awareness of prognosis is from the respondent’s perspective (Supplementary Appendix SA2).

For the 28 patients who died at home (12 died in hospice), the postmortem questionnaire (11 items), created by the research team (Supplementary Appendix SA3), was completed by the HCPs team. The quality of communication during the course of care with the patient and his or her family, the team’s view of symptom control in the last days of life, and the issue of whether there was a concordance between where the patient would have wished to die (if explicit) and the actual place of death were examined. Whether Advance Directives had been drawn up was also taken into consideration.

### Statistical analysis

The questionnaires administered in the study collected only information attributable to qualitative (categorical) variables, which were summarized through absolute and relative frequencies (percentages). Since the questionnaires were administered to the patient every 7–10 days during intake, the assessment of any statistically significant differences between the responses given to the dichotomous categorical variables was evaluated through McNemar’s test for two paired proportions, or through its extension (McNemar-Bowker test) in case there were more than two categories. If more than two questionnaires were administered, the first and last administration, i.e., questionnaires assessing intake and days before death, were evaluated to assess the change in PA over time.

## Results

Forty patients (±75 years old, 60% females) were enrolled; most were geriatric patients. The analysis of the sample showed that the disease determining the patient’s caregiving was predominantly oncological in nature (92%), and the diagnosis of the disease leading to death had been made very recently (≤six months) for 12 patients (30%).

The caregiver (70% females) in all cases was a family member. Fifty percent of them were between 45 and 64 years old, while 45% were over 65 years old (Table 1).

**Table 1.** Social and Clinical Factors of Patient and Caregiver

Patient characteristics	*N* = 40
Age	
** **Median (IQR)	75 (67, 82)
Sex	
M	16 (40%)
F	24 (60%)
Educational level	
Elementary/middle school	17 (42%)
High school diploma	16 (40%)
Bachelor’s degree	7 (28%)
Marital status	
Single	3 (7%)
Married	26 (65%)
Widowed	10 (25%)
Separated	1 (3%)
Employment	
Unemployed	6 (15%)
Unskilled worker	21 (52%)
Skilled office worker	9 (22%)
Manager, entrepreneur	4 (11%)
Significant life-threatening disease type	
Oncology	37 (92,5%)
Cardiovascular	1 (2.5%)
Pulmonological	1 (2.5%)
Infectious	0 (0%)
Neurological	0 (0%)
Nephrological	0 (0%)
Other	1 (2.5%)
Months elapsed from diagnosis to admission of patient	
Median (iqr)	22 (4, 36)
** ≤**Six months	12 (30%)
Reporting origin	
Oncology	21 (53%)
General medicine	7 (17%)
Pulmonary disease	1 (2.5%)
Surgery	1 (2.5%)
Geriatrics	1 (2.5%)
Self-presentation	2 (5%)
Hospice	1 (2.5%)
Mmg	5 (12.5%)
Infectious	1 (2.5%)
Reason for end of treatment	
Death at home	28 (70%)
Hospice transfer	12 (30%)
Karnofsky performance status	
Median (IQR)	40 (40, 50)
Barthel index	
Median (IQR)	83 (71, 100)
Anamnesy of depression	
No	30 (75%)
Yes	10 (25%)
Patient is a believer	
No	9 (23%)
Yes	31 (77%)
Patient is a practicing believer	
No	18 (45%)
Yes	22 (55%)
Caregiver characteristics	
Sex	
M	12 (30%)
F	28 (70%)
Age	
** ≤**44	2 (5%)
45–64	20 (50%)
** ≥**65	18 (45%)
Caregiver’s relationship with the patient	
Spouse/partner	21 (53%)
Son/daughter	16 (40%)
Other family member	3 (7%)
Caregiver’s educational level	
Elementary/middle school	14 (36%)
High school diploma	13 (32%)
Bachelor’s degree	13 (32%)
Time available for caregiver (hour/day)	
Median (IQR)	24.0 (12.0, 24.0)
Duration of intake (days)	
Median (IQR)	50 (29, 90)
*N* (%)	

IQR, interquartile range.

### Physician questionnaire

The study was conducted in a home PC setting. It was found that 85% of patients were aware of the palliative purpose of treatment throughout the home care process.

At the time of admission, it appeared that 30 patients (75%) had previously asked HCPs to be informed about their prognosis. The questionnaire results showed that only 23 patients (57%) were informed of the prognosis by the hospital physician when it could be verified (20, 87%). Regarding the awareness of prognosis at the time of intake by the PC Service, 17 patients (42%) were fully aware of their prognosis, and 4 patients (10%) had not been informed but perceived their terminally ill condition. The total percentage of patients who were aware from the beginning was therefore 52%.

Before death, it appeared that 33 patients (82%) had asked to be informed about their prognosis and 29 (72%) patients were informed about their prognosis. This is statistically significant: the percentage of informed patients increased by 57% to 72%; six patients were informed of their prognosis during the treatment.

Before death, 25 patients (62%) were fully aware of their prognosis, and 4 patients (10%) had not been informed but perceived their terminal condition; and the total percentage of fully aware patients was therefore 72%. This figure is statistically significant because the number of fully aware patients increased during treatment by 20% (*p* value 0.008).

It should be noted that 32 patients out of 40 responders (80%) were already fully aware of diagnosis at the beginning of intake (Table 2). Prior to death, 35 patients (88%) were aware.

**Table 2.** The Medical Questionnaire

Physician questions	Time of intake *N* = 40	Predeath *N* = 40	*p* Value^a^
Do you feel that the patient is aware of the diagnosis and/or more generally of the state of their disease?			NS
** **Yes, the patient is aware	32 (80%)	35 (88%)	
** **The patient fails to internalize	3 (7.5%)	0 (0%)	
** **The patient has not been informed	2 (5.0%)	3 (7.5%)	
** **No, the patient is not aware	3 (7.5%)	2 (5.0%)	
Do you think the caregiver understands the patient’s diagnosis correctly?			NS
** **No	1 (2.5%)	0 (0%)	
** **Yes	39 (98%)	40 (100%)	
Did the patient ask to be informed about his/her diagnosis?			NS
** **No	5 (12%)	4 (10%)	
** **Yes	35 (88%)	36 (90%)	
If yes, when was the diagnosis communicated to the patient?			NS
** **At the time of diagnosis	30 (88%)	30 (86%)	
** **Subsequently	2 (5.9%)	2 (5.7%)	
** **At the time of the intake of the CP service	2 (5.9%)	3 (8.6%)	
** **Unknown	6	5	
If yes, by whom was the patient informed?			NS
** **Hospital physician	30 (88%)	30 (86%)	
** **GP	0 (0%)	0 (0%)	
** **Family member	3 (8.8%)	3 (8.6%)	
** **Other	1 (2.9%)	2 (5.7%)	
** **Unknown	6	5	
In which setting was the patient informed?			NS
** **Hospital ward	21 (62%)	21 (58%)	
** **Outpatient clinic	10 (29%)	11 (31%)	
** **Home	3 (8.8%)	4 (11%)	
** **Other	0 (0%)	0 (0%)	
** **Unknown	6	4	
Did the patient want to be informed about their prognosis?			NS
** **No	10 (25%)	7 (18%)	
** **Yes	30 (75%)	33 (82%)	
Has the prognosis been communicated to the patient?			0.041
** **No	17 (42%)	11 (28%)	
** **Yes	23 (57%)	29 (72%)	
If yes, when was the prognosis communicated to the patient?			NS
** **When it could be verified	20 (87%)	22 (76%)	
** **At the time of admission to cp	3 (13%)	6 (21%)	
** **Other	0 (0%)	1 (3.4%)	
** **Unknown	17	11	
If yes, by whom was the patient informed?			NS
** **Hospital physician	20 (87%)	22 (76%)	
** **GP	0 (0%)	1 (3.4%)	
** **Family member	1 (4.3%)	1 (3.4%)	
** **Other (CP team)	2 (8.7%)	5 (17%)	
** **Unknown	17	11	
Where was the patient informed?			NS
** **Hospital ward	13 (57%)	14 (48%)	
** **Outpatient clinic	6 (26%)	7 (24%)	
** **Home	3 (13%)	7 (24%)	
** **Other	1 (4.3%)	1 (3.4%)	
** **Unknown	17	11	
Do you think the patient is aware of the prognosis?			0.008
** **The patient is fully aware	17 (42%)	25 (62%)	
** **The patient has not been informed but perceives the condition to be terminal	4 (10%)	4 (10%)	
** **The patient has been informed but has unrealistic expectations/underestimates the prognosis	8 (20%)	5 (12%)	
** **The patient is not aware of the prognosis	11 (28%)	6 (15%)	
Do you think the caregiver is aware of the patient’s prognosis?			0.007
** **The caregiver is fully aware	21 (52%)	29 (72%)	
** **The caregiver has not been informed but perceives the terminal prognosis	1 (2.5%)	1 (2.5%)	
** **The caregiver has been informed but has unrealistic expectations/underestimates the prognosis	14 (35%)	9 (22%)	
** **The caregiver is not aware of the prognosis	4 (10%)	1 (2.5%)	
What does the patient think about his or her future?			NS
** **The patient has unrealistic expectations (years)	14 (35%)	7 (18%)	
** **The patient believes that he still has some time to live (months)	21 (52%)	22 (55%)	
** **The patient thinks he will die soon (days)	1 (2.5%)	6 (15%)	
** **Does not express himself	4 (10%)	5 (12%)	
What does the patient think about his or her disease?			NS
** **The patient believes that he/she can be cured	2 (5.0%)	2 (5.0%)	
** **The patient is uncertain about possible recovery	15 (38%)	7 (18%)	
** **The patient thinks he/she will die from this pathology	18 (45%)	29 (72%)	
** **Does not express himself	5 (12%)	2 (5.0%)	
Does the patient talk openly about their condition?			NS
** **No	6 (15%)	4 (10%)	
** **Yes	34 (85%)	36 (90%)	
If yes, with whom?			NS
** **Only health care professionals	5 (15%)	5 (15%)	
** **Health care professionals and family members	23 (67%)	23 (67%)	
** **Health care professionals, family members, friends, caregiver	3 (9%)	3 (9%)	
** **Family members only	3 (9%)	3 (9%)	
** **Unknown	6	6	
Are you aware that you are in hospice/palliative care ward/ucp-dom care?			NS
** **No	6 (15%)	6 (15%)	
** **Yes	34 (85%)	34 (85%)	

Time (days) elapsed between physician questionnaire completion at intake and discharge: Median: 22; IQR: 10.75–40.5.

^a^ McNemar’s chi-squared test with continuity correction; Wilcoxon signed rank test with continuity correction.

GP, general practitioner; NS, not significant.

### Caregiver questionnaire

Regarding prognosis awareness at the time of intake, caregivers reported that 26 patients (65%) were aware of their prognosis. At the time of intake, it was found that 31 patients (78%) had previously asked HCPs to be informed about their prognosis (Table 3).

**Table 3.** The Caregiver Questionnaire

**Caregiver questions**	*N* = 40	*N* = 40
Do you feel that the patient is aware of the diagnosis /or more generally of the state of their disease?		
Yes, the patient is fully aware	29 (72%)	29 (72%)
Yes, the patient knows the main diagnosis but does not understand in detail	6 (15%)	6 (15%)
The patient has not been informed but thinks he/she has an incurable disease	0 (0%)	0 (0%)
No, the patient is not aware	5 (12%)	5 (13%)
Did the patient want/want to know about his/her diagnosis?		
No	3 (7.5%)	3 (7.7%)
Yes	37 (92%)	37 (92%)
If yes, when was the diagnosis communicated?		
At the same time the diagnosis was made	32 (91%)	32 (91%)
Subsequently	3 (8.6%)	3 (8.8%)
Unknown	5	5
If yes, by whom was the patient informed?		
Hospital physician	32 (91%)	32 (94%)
GP	0 (0%)	0 (0%)
Family member	2 (5.7%)	1 (2.9%)
Other	1 (2.9%)	1 (2.9%)
Unknown	5	6
Where?		
Hospital ward	22 (63%)	22 (65%)
Outpatient clinic	10 (29%)	8 (24%)
Home	3 (8.6%)	4 (12%)
Other	0 (0%)	0 (0%)
Unknown	5	6
Did the patient want to be informed about his/her prognosis?		
No	9 (22%)	9 (21%)
Yes	31 (78%)	31 (79%)
Do you think the patient is aware of the prognosis?		
The patient is fully aware	22 (55%)	22 (56%)
The patient has not been informed but perceives their condition	4 (10%)	6 (15%)
The patient has been informed but has unrealistic expectations/underestimates the prognosis	4 (10%)	4 (10%)
The patient is not aware of the prognosis	10 (25%)	7 (18%)
Unknown		1
What does the patient think about his or her future?		
The patient believes that he/she can recover	0 (0%)	1 (2.6%)
The patient is uncertain about possible recovery	18 (45%)	11 (28%)
The patient believes he/she will die	14 (35%)	21 (54%)
The patient does not express an opinion	8 (20%)	6 (15%)
Unknown		1
Has the prognosis been communicated to the patient?		
No	18 (45%)	12 (31%)
Yes	22 (55%)	27 (69%)
Unknown		1
If yes, by whom was the patient informed?		
Hospital physician	21 (91%)	23 (85%)
GP	0 (0%)	0 (0%)
Family member	1 (4.5%)	1 (3.7%)
Other	0 (0%)	3 (11%)
Unknown	18	13
Where was the patient informed?		
Hospital ward	17 (77%)	18 (67%)
Outpatient clinic	4 (18%)	4 (15%)
Home	0 (0%)	4 (15%)
Other	1 (4.5%)	1 (3.7%)
Unknown	18	13
Does the patient talk openly about their condition?		
No	8 (20%)	6 (15%)
Yes	32 (80%)	33 (85%)
Unknown		1
If yes, with whom?		
Health care providers only	2 (6%)	1 (3%)
Health care professionals, family members	22 (67%)	25 (73%)
Health care professionals, family members, friends, caregiver	4 (12%)	3 (9%)
Family members only	5 (15%)	5 (15%)
Unknown	7	6
Is the patient aware that he/she is in a hospice/palliative care ward/ucp-dom care?		
No	6 (15%)	6 (16%)
Yes	34 (85%)	32 (84%)
Unknown		2
What kinds of changes does the diagnosis entail for the patient?		
Well-being	37 (92%)	36 (90%)
Economic	9 (22%)	9 (22%)
Existential	23 (57%)	22 (55%)
Psychosocial	24 (60%)	26 (65%)

Time (days) elapsed between physician questionnaire completion at intake and discharge: Median: 19; IQR: 13–39.75.

The total number of patients aware of prognosis from the caregiver’s perspective before death was 28 (71%).

In reference to the burdens endured by caregivers, defined as those aspects with a psychosocial and physical nature that influenced their existential status (Meffert), at intake and before death they were found to be unchanged over time. The most felt were those burdens regarding assistance (>90%), followed by those of a psychosocial (60%) and existential nature (55%). Economic burdens were perceived as important by 22% of caregivers.

### Postmortem questionnaire

The postmortem questionnaire showed that the HCP team was able to talk freely with the patient about his or her prognosis in 86% of cases and communication was perceived by HCPs as collaborative and effective in 93% of cases.

From the postmortem questionnaire (Table 4), a finding emerged which although limited by the small sample, is relevant: 75% of the patients were explicit regarding the setting where they would have preferred to die, and death in all these cases occurred in the desired place.

**Table 4.** The Postmortem Questionnaire

Post-mortem questions	*N* = 28
Did the patient generate Advance Directives?	
Yes	1 (4%)
No	27 (96%)
If yes, when?	
Before being taken into palliative care	1 (100%)
During the admission to palliative care	0
Did the physician create a Shared Care Planning with the patient?	
Yes	0
No	28 (100%)
If yes, when?	
Before being taken into palliative care	—
During the admission to palliative care	—
How was team-family communication?	
Absent	—
Conflicted	1 (4%)
Problematic	4 (14%)
Cooperative	11 (39%)
Effective	12 (43%)
How was team-patient communication?	
Absent	—
Conflicted	—
Problematic	2 (7%)
Cooperative	11 (39%)
Effective	15 (54%)
How would you define symptom control in the last days of the patient’s life?	
Poor	2 (7%)
Sufficient	5 (18%)
Effective	21 (75%)
Were you able to talk openly with the patient about his or her prognosis?	
Yes	24 (86%)
No	4 (14%)
How much time was spent in moments of communication with the patient?	
Sufficient	16 (57%)
More than usual	10 (36%)
Less than usual	2 (7%)
Did the patient express explicitly where he/she would prefer to die?	
Yes	21 (75%)
No	7 (25%)
If yes, did the death occur in the desired setting?	
Yes	21 (100%)
No	—

The results show that only one patient had drafted Advance Directives.

## Discussion

Regarding the home PC service, it is likely that the home setting itself influences patients’ awareness of prognosis, as there is evidence that understanding the palliative aim of treatment is a proxy for awareness of prognosis.^5^

With regards to prognosis awareness,^17,18^ about half of the patients’ caregivers were aware at the time of PC service intake.

The percentage of patients aware of prognosis prior to death appears to be higher than that indicated at the time of admission.

This is in line with what has been reported by other studies^1,19^ that have analyzed prognosis awareness along the course of care. As Hsiu Chen et al. suggested,^9^ the degree of awareness tends to remain stable or to increase along the course of care in those patients who have poor prognosis awareness at intake.

Therefore, it is crucial that we be able to track the data on prognosis awareness in these patients, as it seems to increase in the moments just before death.

Increased awareness, as a dynamic process, is useful for greater patient involvement in clinical EOL scripts.^20–23^ This finding underscores the HCPs’ role in accompanying the patient in this evolution toward awareness of their terminal state.

It was found that thorough communication regarding prognoses would facilitate a shared clinical and therapeutic pathway and increase the degree of satisfaction with the care provided at the end of life.^24–26^

The awareness of diagnosis promotes communication regarding prognosis in most patients (80%) and almost all caregivers (98%).

This study shows a higher prevalence of prognosis awareness than reported in the literature.^8^

This may testify to cultural evolution in dealing with EOL issues, with health care providers who are more willing to deal with difficult communication in a timely fashion, and on the other hand, citizens who are more desirous of being informed about the evolution of their disease.

The physician’s and caregiver’s positions regarding patient awareness of prognosis are notably similar along the care pathway.^19,26–28^

The literature review shows how the role of the caregiver is critical in investigating the patient’s awareness, and in particular how it evolves along the course of care.^12,29,30^

Some studies use the caregiver’s point of view as an integral factor in assessing the patient’s awareness about his or her condition.^19,28,31^ In this study, the caregiver questionnaire turns out to partly mirror the one administered to the physician so that two different but complementary viewpoints about awareness of prognosis are obtained. The data that refer to the caregiver confirm what emerged from the physician questionnaire about the settings and modes of communication of prognosis which occurred along the course of the disease.

The questionnaire asked what burdens the caregiver believed to be his/her responsibility.^13,32,33^ The caregiver’s activity appears to be a significant commitment, considering that more than 55% were engaged 24/24 hours; it is therefore reasonable to expect that a significant impact on the caregiver’s quality of life was felt. The questionnaire showed that most caregivers took responsibility for all aspects of the assistance they were required to provide, and economic burdens were found to be insignificant, as they were actually perceived by only 20% of caregivers.

A positive experience regarding home care was reported by the HCP team in most cases, both regarding the family and the patient.

In line with the literature,^5,34,35^ from the postmortem questionnaire it also emerged that the experience of communication was viewed as positive by the team; a true therapeutic alliance was created along the journey of the care of the terminally ill patient. Communication between team and family was reported to be collaborative and effective in 82% of cases (no. 23), and between team and patient in 92% of cases (no. 26).

Despite the patient’s lack of awareness of their prognosis at the time of intake, the patient’s home care pathway allowed the various professionals involved to explore what the patient’s perceptions of their disease and of their future were with them. This communication process was likely made possible due in part to the length of time taken to care for patients at home, which is generally longer in these situations than in hospice.

A finding emerged from the postmortem questionnaire, which although limited by the small sample, is relevant: 75% of patients explicitly expressed where they would prefer to die, and death in all these cases occurred at the desired location. There are few studies that explore place of death preference, most of which were conducted on samples of healthy people. Clearly, exploring this preference in a sample of sick patients, mostly aware that they are suffering from incurable disease a few weeks before their death, has a different relevance. It is particularly significant that this preference was explicitly expressed and was subsequently respected during treatment.

The results show that only one patient had drafted Advance Directives; this finding confirms how the drafting of Advance Directives, an important tool not only because they allow future health care treatments to be adjusted to the wishes of patients but also because they reduce the risk of inadequate or unnecessary treatments, is still an underutilized practice, perhaps in part because it is not well known.

## Limitations of the Study

The study has several limitations: first, the small number of patients. It is hoped that research can develop multicenter studies on this issue, including international studies to analyze experiences in different sociocultural contexts.

In addition, the questionnaires used were not validated but can be generalizable and used at home PC services. In this study, a semi-structured method was chosen to investigate prognosis awareness. However, it would be desirable to develop validated scores, which as of today are not yet available.

A further limitation concerns the nonadministration of the questionnaire investigating prognosis awareness to other HCPs. This is because in the Italian context, the responsibility for communicating diagnosis and prognosis is medical. In other countries, the responsibility may fall on other HCPs.

## Conclusions

During the PC phase, awareness of the prognosis increased significantly from the time of intake.

Prognosis awareness, in addition to having important clinical implications, is also relevant to the patient and his/her caregivers. Indeed, prognosis awareness enables informed choices about issues, which must be faced in a timely fashion.

More effective communication related to prognosis would facilitate a shared clinical and therapeutic pathway, increasing the degree of satisfaction with the care provided at the end of life.

In particular, given the findings of our study, we hope for increasing and homogenous growth in the awareness of the importance of this issue, especially throughout our country.

The views of the physician and caregiver regarding the patient’s awareness of prognosis are notably similar along the care pathway.

In most cases, the HCP team reported a positive experience regarding home care, both with the family and the patient.

Open, collaborative, and effective communication is a key prerequisite to the fact that a significant percentage of patients were able to explicitly describe the setting where they would prefer to die, and death in all of these cases occurred at the desired location.

## Notes

Supplementary Appendix SA1

Supplementary Appendix SA2

Supplementary Appendix SA3

## Abbreviations Used

HCPs: healthcare professionals
PC: palliative care
