# Supplementary material for: What Have You Been Told? Awareness of Prognosis of Patients in an Italian Home Palliative Care Service
Source: Palliat Med Rep. 2025 Feb 10;6(1):17–27. doi: 10.1089/pmr.2024.0072 (PMC11959208; doi:10.1089/pmr.2024.0072)
Supplement: Supplementary Appendix SA1 [file pmr.2024.0072_supp_appendix_sa1.docx]

**Appendix A**

**Questionnaire filled out by the doctor.**

| 1.Do you think the patient is aware of the diagnosis and/or more generally of the state of his/her disease?  □ Yes, the patient is aware of the diagnosis(s) although not in detail (e.g., metastasis/stage of disease, outcome of investigations and therapies)  □ The patient fails to internalize the diagnosis that has been communicated  □ The patient has not been informed but perceives the existence of a life-threatening condition  □ no, the patient is not aware  2.Do you think the caregiver correctly understands the patient's diagnosis?  □ yes  □ no  3.Has the patient asked to be informed regarding his/her diagnosis?  □ yes  □ no  4.If yes, when was the diagnosis communicated?  □ at the time the diagnosis was made  □ subsequently  □ at the time the CP service took over  5.If yes, by whom was the patient informed?  □ hospital physician  □ GP  □ family member  □ other (specify ...............)  6.Where was the patient informed?  □ in a hospital ward  □ in an outpatient clinic  □ at home  □ other (specify ...............)  7.Did the patient desire to be informed about his/her prognosis?  □ yes  □ no  8.Was the prognosis communicated to the patient?  □ yes  □ no  9.If yes, when was the prognosis communicated to the patient?  □ when it could be verified  □ at the time of admission to CP  □ other (specify .......................)  10. If yes, by whom was the patient informed?  □ hospital physician  □ GP  □ family member  □ other (specify .......................)  11. Where was the patient informed?  □ in a hospital ward  □ in an outpatient clinic  □ at home  □ other (specify .......................)  12. Do you think the patient is aware of the prognosis?  □ the patient is fully aware  □ the patient has not been informed but perceives their condition to be terminal  □ the patient has been informed but has unrealistic life expectations/underestimates the prognosis  □ the patient is not aware of the prognosis  13. Do you think the caregiver is aware of the patient's prognosis?  □ yes, the caregiver is fully aware of the prognosis  □ yes, the caregiver has not been informed but perceives the patient’s terminally ill condition  □ the caregiver has been informed but has unrealistic life expectations/underestimates the prognosis  □ the caregiver is unaware of the prognosis  14. What does the patient think about his or her future?  □ the patient has unrealistic expectations (years)  □ the patient thinks he or she still has some time to live (months)  □ the patient thinks he/she will die soon (days)  □ does not express himself or herself  15. What does the patient think about his or her condition?  □ the patient believes he/she can get well  □ the patient is uncertain about possible recovery  □ the patient thinks he/she will die from this pathology  □ does not express himself or herself  16. Does the patient talk openly about his or her condition?  □ yes  □ no  17. If yes, with whom?  (more than one answer is possible)  □ health care providers  □ family members  □ friends  □ spiritual guide  □ all of the above  18. Are you aware that you are in hospice/palliative care ward/ UCP-dom charge?  □ yes  □ no  19. Who does the patient designate as his/her primary caregiver?  Name:……………Surname:……………… Address………… |
| --- |
