# Supplementary material for: What Have You Been Told? Awareness of Prognosis of Patients in an Italian Home Palliative Care Service
Source: Palliat Med Rep. 2025 Feb 10;6(1):17–27. doi: 10.1089/pmr.2024.0072 (PMC11959208; doi:10.1089/pmr.2024.0072)
Supplement: Supplementary Appendix SA2 [file pmr.2024.0072_supp_appendix_sa2.docx]

**Appendix B**

**Questionnaire filled out by the caregiver**

| Sex 1st Caregiver | sex M=0, F=1 |
| --- | --- |
| Age 1st caregiver | 0: <44 yo; 1: 45-64 yo; 2: > 65 yo |
| Relationship_1^st^ caregiver to pz | 0: spouse/partner; 1: son/daughter; 2: father/mother; 3: other family member; friend; 4:former spouse |
| Caregiver Education level | 0: elementary/middle school 1: high school diploma 2: college degree |
| Available Time in h/day | … |

| 1.Do you feel that the patient is aware of the diagnosis /or more generally of the state of his/her disease?  □ yes, the patient is fully aware  □ yes, the patient knows the main diagnosis but not in detail (e.g., metastasis/stage of disease, outcome of investigation and treatment)  □ patient has not been informed but thinks he/she has an incurable disease  □ no, the patient is not aware  2. Did the patient want/want to know about their diagnosis?  □ yes  □ no  3.If yes, when was the diagnosis communicated?  □ at the same time the diagnosis was made  □ subsequently  4.If yes, by whom was the patient informed?  □ hospital physician  □ GP  □ family member  □ other (specify …)  5.Where was the patient informed?  □ In a hospital ward  □ in an outpatient clinic  □ at home  □ other (specify …)  6.Did the patient want to know about his/her prognosis?  □ yes  □ no  7.Do you think the patient is aware of the prognosis?  □ the patient is fully aware  □ the patient has not been informed but perceives his/her condition  □ the patient has been informed but has unrealistic life expectations/underestimates the prognosis  □ the patient is not aware of the prognosis  8.What does the patient think about his or her future?  □ the patient believes he/she can get well  □ the patient is uncertain about possible recovery  □ the patient thinks he or she will not make it and will die  □ does not express an opinion about his/her future  9.Has the prognosis been communicated?  □ yes  □ no  10. If yes, when was it communicated?  …/…./…. ……………………………………  11. If yes, by whom was the patient informed?  □ hospital physician  □ GP  □ family member  □ other(specify..........................................)  12. Where was the patient informed?  □ in a hospital ward  □ in an outpatient clinic  □ at home  □ other (specify..........................................)  13. Does the patient talk openly about his or her condition?  □ yes  □ no  14. If yes, with whom (*more than one answer is possible*).  □ health care providers  □ family members  □ friends  □ spiritual guide  □ all of the above  15. are you aware that you are in hospice/palliative care ward/ UCP-dom care?  □ yes  □ no  16. What burdens may be borne by you?  (multiple answers possible)  □ well-being  □ economic  □ existential  □ psycho-social |
| --- |
