# Supplementary material for: What Have You Been Told? Awareness of Prognosis of Patients in an Italian Home Palliative Care Service
Source: Palliat Med Rep. 2025 Feb 10;6(1):17–27. doi: 10.1089/pmr.2024.0072 (PMC11959208; doi:10.1089/pmr.2024.0072)
Supplement: Supplementary Appendix SA3 [file pmr.2024.0072_supp_appendix_sa3.docx]

**Appendix C**

**Staff Post Mortem Questionnaire**

| 1.Did the patient generate Advance Directives?  □ yes  □ no  2. If yes, when?  □ before being taken into Palliative Care.  □ during the admission to Palliative Care  3.Did the physician create a Shared Care Planning with the patient ?  □ yes  □ no  4. If yes, when?  □ before being taken into Palliative Care.  □ during the admission to Palliative Care  5. How was team-family communication?  □ absent  □ conflicted  □ problematic  □ cooperative  □ effective  6. How was team-patient communication?  □ absent  □ conflicted  □ problematic  □ cooperative  □ effective  7.How would you define symptom control in the last days of the patient's life?  □ poor  □ sufficient  □ effective  8.Were you able to talk openly with the patient about his or her prognosis?  □ yes  □ no  9. How much time was spent in moments of communication with the patient?  □ sufficient  □ more than usual  □ less than usual  10. Did the patient express explicitly where he/she would prefer to die?  □ yes  □ no  11. If yes, did the death occur in the desired setting?  □ yes  □ no |
| --- |
